# The Ratio of Monocytes to Lymphocytes in Peripheral Blood Correlates with Increased Susceptibility to Clinical Malaria in Kenyan Children

**DOI:** 10.1371/journal.pone.0057320

**Published:** 2013-02-20

**Authors:** George M. Warimwe, Linda M. Murungi, Gathoni Kamuyu, George M. Nyangweso, Juliana Wambua, Vivek Naranbhai, Helen A. Fletcher, Adrian V. S. Hill, Philip Bejon, Faith H. A. Osier, Kevin Marsh

**Affiliations:** 1 The Jenner Institute, University of Oxford, Oxford, United Kingdom; 2 Kenya Medical Research Institute-Wellcome Trust Research Programme, Kilifi, Kenya; 3 Wellcome Trust Centre for Human Genetics, University of Oxford, Oxford, United Kingdom; 4 Centre for Clinical Vaccinology and Tropical Medicine, University of Oxford, Oxford, United Kingdom; Walter & Eliza Hall Institute, Australia

## Abstract

**Background:**

*Plasmodium falciparum* malaria remains a major cause of illness and death in sub-Saharan Africa. Young children bear the brunt of the disease and though older children and adults suffer relatively fewer clinical attacks, they remain susceptible to asymptomatic *P. falciparum* infection. A better understanding of the host factors associated with immunity to clinical malaria and the ability to sustain asymptomatic *P. falciparum* infection will aid the development of improved strategies for disease prevention.

**Methods and Findings:**

Here we investigate whether full differential blood counts can predict susceptibility to clinical malaria among Kenyan children sampled at five annual cross-sectional surveys. We find that the ratio of monocytes to lymphocytes, measured in peripheral blood at the time of survey, directly correlates with risk of clinical malaria during follow-up. This association is evident among children with asymptomatic *P. falciparum* infection at the time the cell counts are measured (Hazard ratio (HR)  =  2.7 (95% CI 1.42, 5.01, P  =  0.002) but not in those without detectable parasitaemia (HR  =  1.0 (95% CI 0.74, 1.42, P  =  0.9).

**Conclusions:**

We propose that the monocyte to lymphocyte ratio, which is easily derived from routine full differential blood counts, reflects an individual's capacity to mount an effective immune response to *P. falciparum* infection.

## Introduction

*Plasmodium falciparum* malaria is still a major cause of morbidity and mortality in sub-Saharan Africa where the greatest burden of disease is borne by young children [1], [2]. Substantial clinical immunity develops following repeated natural exposure to *P. falciparum* such that clinical malaria tends to be less frequent in children over 5 years of age and adults. Despite this, older children and adults remain susceptible to asymptomatic, often chronic, *P. falciparum* infections to which immunity probably never occurs [3], [4]. This distinction between immunity to clinical malaria and immunity against *P. falciparum* infection *per se* is further evident in the epidemiological pattern of clinical malaria and asymptomatic parasitaemia in areas endemic for *P. falciparum* transmission. Whilst the frequency of clinical malaria among individuals resident in these settings tends to be highly seasonal, that of asymptomatic *P. falciparum* infections remains relatively constant throughout the year [3], [4]. Thus, as proposed previously [5], individuals with pre-existing asymptomatic *P. falciparum* infection must often develop clinical malaria perhaps following infection by parasites with different antigenic properties from those associated with the asymptomatic infection. The specific host factors underlying susceptibility to clinical malaria despite the ability to sustain asymptomatic *P. falciparum* infection are poorly understood.

Longitudinal studies in which individuals are recruited at cross-sectional surveys and their risk of developing disease assessed in a defined follow-up period have been a key tool in identifying correlates of immunity to clinical malaria [6]. Several factors, assessed at the time of recruitment to such studies, have been shown to modify an individual's risk of clinical malaria in the follow-up period. These include age, antibodies to certain parasite blood-stage antigens and, carriage of asymptomatic *P. falciparum* infection at the time of survey, among others [2], [7]–[9]. The levels of specific immune responses among children with asymptomatic *P. falciparum* infection at the time of survey have been observed to be higher and to exhibit stronger correlation with reduced risk of disease during follow-up when compared to responses in children without detectable parasitaemia [8], [10]–[12]. These data have highlighted the need for accounting for asymptomatic parasitaemia in studies using a longitudinal framework to identify correlates of immunity to clinical malaria.

Full differential cell counts from peripheral blood are routinely used as an aid to clinical diagnosis of a wide range of infectious diseases, including malaria, where they have been associated with prognosis in several studies [13]–[16]. However, their utility as a correlate of disease risk in longitudinal studies has been poorly investigated. Given the central role of monocytes and lymphocytes in the induction of immune responses, their frequency in peripheral blood might be expected to reflect the state of an individual's immune response to infection. In a recent transcriptional analysis of peripheral blood mononuclear cells from a cohort of South African infants the relative frequency of myeloid-specific to lymphoid-specific transcripts at the start of monitoring was shown to predict risk of developing tuberculosis disease during follow-up (H.A.F. and A.V.S.H., unpublished data). Here, using full differential blood count data from Kenyan children included in five cross-sectional surveys, we sought to determine whether the relative count of monocytes to lymphocytes in peripheral blood (hereafter termed “ML ratio”) can identify children at most risk of developing clinical malaria during follow-up. We find that among children with asymptomatic *P. falciparum* infection at the time their full differential blood counts are measured, high ML ratio is associated with an increased risk of clinical malaria episodes during follow-up.

## Materials and Methods

### Ethics statement

The Kenya Medical Research Institute National Ethical Review committee granted approval for this study. Written informed consent was obtained from parents or guardians of all study participants.

### Study location and population

This study was conducted at Kilifi District Hospital on cohort data from children resident in Junju sub-location of Kilifi district, Kenya, and is part of the Kilifi Health and Demographic Surveillance System [17]. As of 2010, Junju had an estimated entomological inoculation rate of 21.7 [18] though malaria transmission in the wider Kilifi district has been on the decline since 1999 [19]. Cohort data from children included in five annual cross-sectional surveys performed just before the rainy season in May 2007, 2008, 2009, 2010 and 2011, respectively, were used. Blood samples were collected from each individual in the respective surveys and a full differential blood count performed using a Coulter Counter® (Beckman Coulter, Inc.). In addition thick and thin blood smears were made, Giemsa-stained and examined for *P. falciparum* parasites by microscopy. Children that were febrile at the time of survey, whether carrying parasites in peripheral blood (“parasite positive”) or not (“parasite negative”), were treated appropriately by a clinician at the Kilifi District Hospital and excluded from this study. No treatment was administered to asymptomatic parasite positive children. A summary of the numbers of parasite positive and parasite negative children included in this study from each of the five surveys and their respective characteristics are shown in Table 1.

**Table 1:** Summary characteristics of the study population.

Year of survey	Variable	Parasite negative (Median, IQR)	Parasite positive (Median, IQR)
2007	Number sampled	269	50
	Age (years)	4.5 (3.1, 6.4)	5.8 (4.1, 6.6)
	Monocyte count (x10^3^/µl of blood)	0.64 (0.49, 0.86)	0.69 (0.49, 0.85)
	Lymphocyte count (x10^3^/µl of blood)	3.69 (2.87, 4.83)	3.78 (3.12, 4.39)
	ML ratio	0.17 (0.14, 0.21)	0.19 (0.13, 0.22)
2008	Number sampled	226	93
	Age (years)	5.0 (3.2, 7.1)	6.7 (5.1, 7.7)
	Monocyte count (x10^3^/µl of blood)	0.50 (0.37, 0.65)	0.46 (0.37, 0.58)
	Lymphocyte count (x10^3^/µl of blood)	3.26 (2.4, 4.52)	2.65 (2.00, 3.58)
	ML ratio	0.15 (0.12, 0.19)	0.17 (0.13, 0.23)
2009	Number sampled	264	68
	Age (years)	6.0 (3.7, 8.0)	7.2 (5.6, 8.5)
	Monocyte count (x10^3^/µl of blood)	0.59 (0.47, 0.76)	0.65 (0.49, 0.83)
	Lymphocyte count (x10^3^/µl of blood)	3.49 (2.85, 4.75)	3.50 (2.90, 4.25)
	ML ratio	0.16 (0.13, 0.21)	0.19 (0.15, 0.23)
2010	Number sampled	242	85
	Age (years)	6.5 (3.4, 9.1)	7.8 (6.6, 9.2)
	Monocyte count (x10^3^/µl of blood)	0.60 (0.47, 0.77)	0.63 (0.50, 0.76)
	Lymphocyte count (x10^3^/µl of blood)	3.44 (2.69, 4.43)	3.26 (2.58, 4.07)
	ML ratio	0.18 (0.14, 0.22)	0.19 (0.15, 0.25)
2011	Number sampled	276	80
	Age (years)	7.2 (3.6, 9.7)	9.2 (7.1, 10.7)
	Monocyte count (x10^3^/µl of blood)	0.61 (0.43, 0.92)	0.64 (0.53, 0.84)
	Lymphocyte count (x10^3^/µl of blood)	3.41 (2.70, 4.44)	3.18 (2.58, 3.88)
	ML ratio	0.18 (0.13, 0.24)	0.20 (0.17, 0.26)

Presented are the medians and interquartile ranges (IQR) for age, monocyte count, lymphocyte count and ML ratio at the time of survey for children recruited in each respective cross-sectional survey. The data are shown by year of survey and are stratified by parasite positive/negative status at the time of survey.

### Monitoring for clinical malaria episodes

Weekly active surveillance was used to monitor episodes of clinical malaria by trained field-workers. In addition, passive surveillance was undertaken by trained field-workers based at villages across the study area and at local dispensaries. Monitoring for parasites was only done in children with a history of fever. ML ratios were not measured at the time of clinical malaria episodes. Detailed surveillance procedures have been published previously [18], [20]. Clinical malaria was defined as fever (axillary temperature ≥37.5 °C) plus any parasite density for children under 1 year old and fever accompanied by parasite density >2500 parasites/µl of blood for all other children [21].

### Statistical analysis

All analyses were done in Stata™ version 11 and P value <0.05 used as the cut-off for statistical significance. Negative binomial regression models were used to estimate the associations between clinical malaria episodes and explanatory variables, offset by the period of observation during which children were at risk of clinical malaria. The risk period was defined as the period between date of recruitment to cross-sectional survey and either the date when an individual was lost to follow-up or 31^st^ December 2011. Observed associations were then confirmed using unadjusted Cox regression models to estimate the relationship between explanatory variables and time to first clinical malaria episode. Where data from all five cohorts were considered in the Cox regression modeling, ML ratio or the respective cell count data were included as time-varying covariates.

The May 2008 cross-sectional survey was used as the baseline survey for the primary analysis since children sampled at this time point had antibody data available, allowing adjustment for known antibody correlates of immunity to malaria. First, full differential blood count data from the 319 children sampled at the May 2008 baseline survey were used to estimate the association between ML ratio and total number of clinical malaria episodes experienced during the period between sampling at the May 2008 baseline survey and either the date when an individual was lost to follow-up or 31^st^ December 2011. Then, the effect of adjustment for age and antibodies to the parasite blood-stage antigens apical membrane antigen 1 (AMA1) and merozoite surface proteins 2 (MSP2) and 3 (MSP3), respectively, was examined. Finally, to assess the temporal stability of observed associations between ML ratio and risk of clinical malaria, full differential blood count data from children sampled in the May 2008 survey (N  =  319) or in any of four other cross-sectional surveys conducted in May 2007 (N  =  319), 2009 (N  =  332), 2010 (N  =  327) and 2011 (N  =  356), respectively, were used. The number of clinical malaria episodes in the respective inter-survey periods (that is, between May 2007 and May 2008, May 2008 and May 2009, May 2009 and May 2010, May 2010 and May 2011 and, May 2011 and 31^st^ December 2011) were used as the dependent variable in the negative binomial regression modeling while accounting for: i) multiple sampling of individuals recruited into more than one survey using the robust sandwich estimator, ii) the year of survey as a fixed effect and, iii) age at each survey as a continuous variable.

## Results

A total of 762 episodes of clinical malaria (range, 0 – 6 per child) were experienced over the entire duration of the study, that is, between survey at May 2007 and 31^st^ December 2011. Parasite positive children accounted for fewer of these episodes (121 compared to 641 in parasite negative children). In a negative binomial model predicting frequency of clinical malaria episodes using parasite positive/negative status while accounting for age, year of survey and multiple sampling of individuals, carriage of asymptomatic *P. falciparum* infection at survey was associated with reduced risk of clinical malaria (Incidence rate ratio (IRR)  =  0.7, 95% confidence interval (CI) 0.56, 0.85, P  =  0.0004).

### ML ratio at baseline survey is positively associated with risk of clinical malaria during follow-up

To assess the relationship between ML ratio and risk of clinical malaria we first used the May 2008 survey as baseline since antibody data were available for children sampled at this time, allowing adjustment for known antibody correlates of immunity to malaria. Whereas all models were adjusted for age, associations were first estimated before adjustment for antibodies and the effect of this adjustment assessed thereafter.

**Figure 1:**
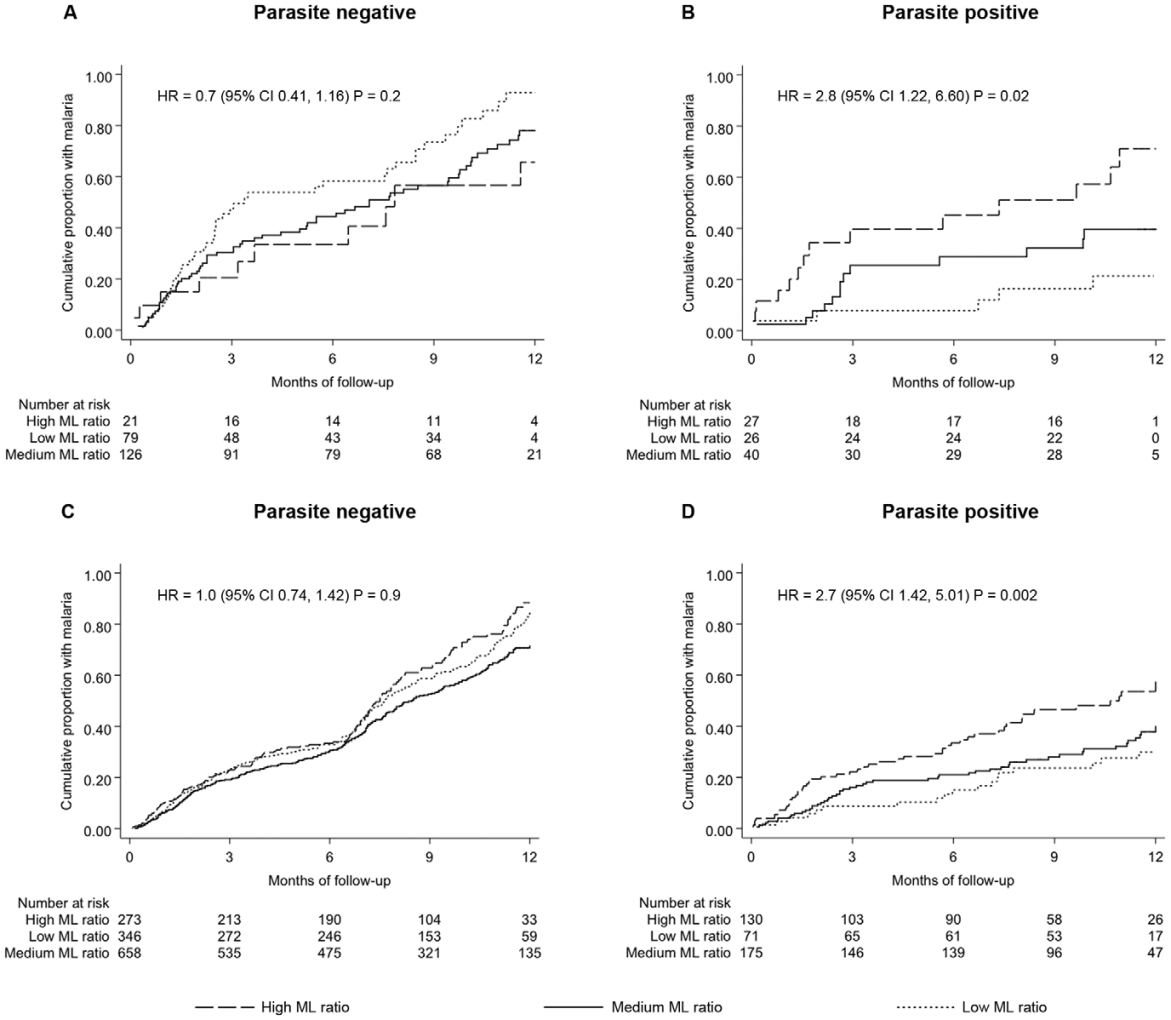
ML ratio positively correlates with risk of clinical malaria. Kaplan-Meier plots of the relationship between ML ratio and time to first episode of clinical malaria during follow-up is shown. (A) and (B) represent results using ML ratios measured in the May 2008 baseline survey and consider a follow-up period ending on 31^st^ December 2011. However, most parasite positive children had experienced their first clinical malaria episode within a year since sampling in the May 2008 baseline survey and so the plots show data for the first 12 months of follow-up. (C) and (D) represent results based on ML ratios measured at each of five surveys (May 2007, 2008, 2009, 2010 and 2011) and consider time to the first episode within the respective one year inter-survey periods as the primary endpoint. The hazard ratios (HR) from unadjusted Cox regression models using ML ratio as the only explanatory variable are shown. The cumulative proportion of children with malaria in relation to their ML ratio, stratified into three arbitrary groups, is shown. “High ML ratio” and “Low ML ratio” represent children whose ML ratio falls in the top and bottom 25^th^ percentile of the sampled population, respectively, whilst “Medium ML ratio” represents all other children.

High ML ratios were associated with high frequency of clinical episodes among children parasite positive at baseline (IRR  =  2.2, 95%CI 1.10, 4.50, P  =  0.03; unadjusted for antibodies) but no association was observed among children parasite negative at baseline (IRR  =  0.8, 95% CI 0.58, 1.17, P  =  0.3; unadjusted for antibodies). Survival analysis using time to first episode as the primary endpoint confirmed the association between high ML ratio and risk of clinical malaria (Fig. 1A–B). Furthermore, the interaction between parasite positive/negative status at baseline and ML ratio was found to be statistically significant (IRR  =  2.6, 95%CI 1.28, 5.40, P  =  0.008; unadjusted for antibodies).

There was no correlation between ML ratio and age at recruitment among parasite positive (rho  =  −0.05, P  =  0.6) or among parasite negative children (rho  =  0.06, P  =  0.4). We considered the possibility that the association between ML ratio and disease might act through an association between ML ratio and protective antibodies to certain *P. falciparum* blood-stage antigens [8]. If this were the case we would predict that the association between ML ratio and total episode count would not be significant following adjustment for antibody levels. Total IgG antibody data, measured using a published enzyme-linked immunosorbent assay protocol [8], from 288 children sampled at the same time as the ML ratio data in the May 2008 baseline survey were available for the blood-stage antigens AMA1, MSP2 and MSP3, respectively. Antibodies to AMA1 and MSP3, but not MSP2, correlated with reduced risk of clinical malaria episodes (Table 2). However, adjusting for these antibody variables did not confound the association between high ML ratio and frequent episodes of clinical malaria (Table 2). The independence of these antibody measures and ML ratio in predicting risk of malaria suggests that ML ratio modulates risk of clinical malaria through a mechanism that is distinct from antibody production.

**Table 2:** The association between risk of malaria and ML ratio is independent of age and antibodies to parasite blood-stage antigens.

		PARASITE NEGATIVE (N = 207)		PARASITE POSITIVE (N = 81)	
Analysis type	Variable	IRR (95% CI)	P value	IRR (95% CI)	P value
Univariate	ML ratio	0.8 (0.55, 1.17)	0.3	2.8 (1.48, 5.12)	0.001
	Age	0.9 (0.88, 0.96)	0.0002	0.8 (0.69, 0.96)	0.02
	AMA1 antibodies	0.3 (0.17, 0.64)	0.001	0.2 (0.10, 0.55)	0.0007
	MSP2 antibodies	1.4 (1.01, 1.81)	0.04	0.8 (0.46, 1.56)	0.6
	MSP3 antibodies	0.8 (0.29, 2.01)	0.6	0.3 (0.17, 0.69)	0.002
	Parasite schizont extract	1.5 (1.20, 1.78)	0.0001	1.2 (0.72, 2.02)	0.5
Multivariate	ML ratio	0.9 (0.61, 1.20)	0.4	2.2 (1.16, 4.28)	0.02
	Age	0.9 (0.87, 0.96)	0.0002	0.8 (0.75, 0.96)	0.01
	AMA1 antibodies	0.4 (0.17, 0.91)	0.03	0.3 (0.13, 0.83)	0.02
	MSP2 antibodies	1.1 (0.75, 1.49)	0.7	1.1 (0.58, 2.09)	0.8
	MSP3 antibodies	0.5 (0.26, 1.08)	0.08	0.4 (0.22, 0.85)	0.01
	Parasite schizont extract	1.7 (1.35, 2.04)	0.000002	1.5 (1.00, 2.40)	0.05

Presented are incidence rate ratios (IRR) and 95% confidence intervals (CI) from negative binomial regression models predicting the total number of malaria episodes between sampling at May 2008 baseline survey and 31^st^ December 2011 using ML ratio, age, antibodies to AMA1, MSP2 and MSP3 and to parasite schizont extract, used routinely as a control for previous parasite exposure in antibody assays [8]. Univariate analysis is done using each variable in turn whereas all variables are included in the multivariate model and the respective results from each variable shown. As with the complete dataset on which the antibody data is based a statistically significant interaction between parasite positive/negative status at baseline and ML ratio was evident (IRR  =  2.7, 95%CI 1.32, 5.47, P  =  0.006).

### The association between high ML ratio and risk of clinical malaria is stable over time

**Figure 2:**
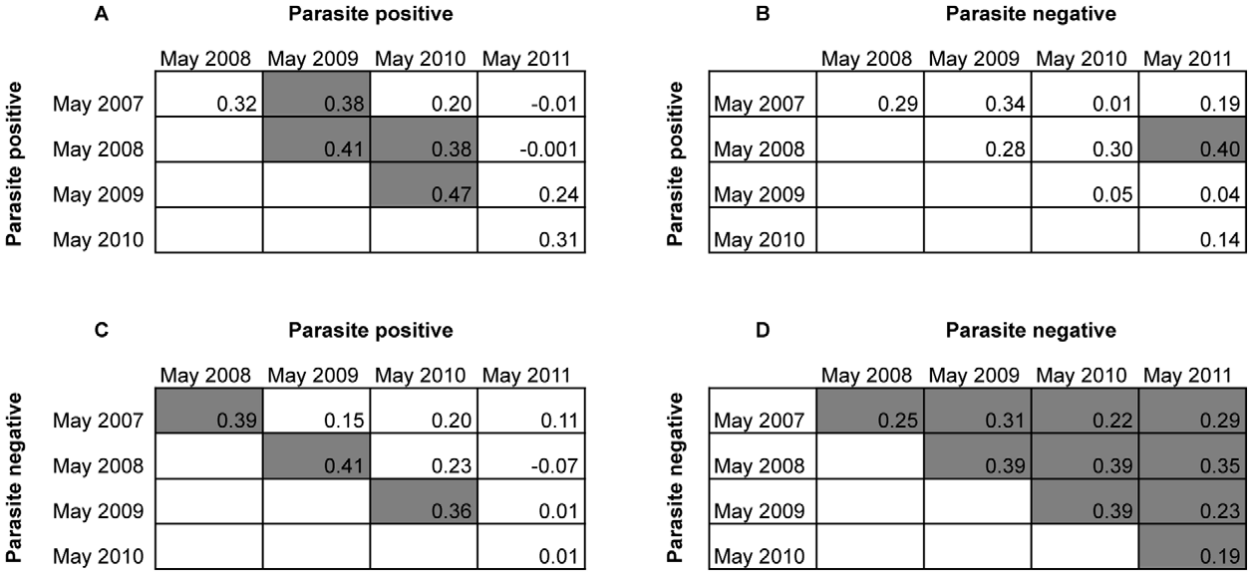
Comparison between ML ratios measured at different cross-sectional surveys. Spearman rank correlation coefficient is used to assess the relationship between ML ratios across different surveys according to parasite positive/negative status at the time the ML ratio was measured. Results are shown for children that were parasite positive at the May 2007 or 2008 or 2009 or 2010 survey and parasite positive (A) or parasite negative (B) in subsequent surveys (that is 2008–2011). In (C) and (D) results are shown for children that were parasite negative at the May 2007 or 2008 or 2009 or 2010 survey and parasite positive (C) or parasite negative (D) in subsequent surveys (that is 2008–2011). Rho values from all comparisons are shown and statistically significant comparisons (P<0.05) indicated in shaded boxes. Unshaded boxes represent comparisons that showed no significant correlation.

We next investigated the relationship between ML ratio and risk of clinical malaria over time using both the ML ratios measured at the May 2008 baseline survey and those measured at each of four other surveys carried out in May 2007, 2009, 2010 and 2011, respectively. Overall, the range of ML ratios measured at the different surveys was comparable across the five surveys (Table 1). However, a positive correlation of ML ratios between different surveys was only observed consistently when comparisons were made between parasite negative children (Fig. 2) suggesting that ML ratios are stable over time but only in the absence of *P. falciparum* infection. Despite the statistical significance among parasite negative children the Spearman's rank correlation coefficients were relatively low (all less than 0.50, Fig. 2). This might be explained if some of the children classified as parasite negative at cross-sectional survey had an asymptomatic *P. falciparum* infection shortly before sampling at the survey, which perturbed their ML ratio. However, monitoring for asymptomatic *P. falciparum* infections was not done in this study and thus no firm conclusions can be drawn regarding this possibility. Further, since we used light microscopy rather than PCR-based parasite detection methods it is plausible that some of the parasite negative children might have had asymptomatic *P. falciparum* infection at very low parasitaemia, below the detection limit of light microscopy. Future studies monitoring asymptomatic *P. falciparum* infections by the more sensitive PCR methods [22], [23] will help provide a better assessment of the extent to which ML ratios fluctuate over time among children living in areas endemic for *P. falciparum.*

To test whether the observed association between high ML ratio and clinical malaria is stable over time we used the number of clinical malaria episodes in the respective inter-survey periods (that is, between May 2007 and May 2008, May 2008 and May 2009, May 2009 and May 2010, May 2010 and May 2011 and, May 2011 and 31^st^ December 2011) as a dependent variable in the negative binomial regression modeling while accounting for i) multiple sampling of individuals recruited into more than one survey using the robust sandwich estimator, ii) the year of survey as a fixed effect and, iii) age at each survey as a continuous variable. A positive association between ML ratio and risk of multiple episodes of malaria was evident among children that were parasite positive at the time of measuring their ML ratio (IRR  =  2.0, 95%CI 1.27, 3.20, P  =  0.003). No such association was observed in parasite negative children (IRR  =  1.1, 95%CI 0.86, 1.30, P  =  0.6). These findings were again confirmed by survival analysis using time to first episode as an endpoint (Fig. 1C–D). The results thus suggest that the relationship between ML ratio in children with an ongoing asymptomatic *P. falciparum* infection and susceptibility to frequent episodes of malaria is stable over time.

### Monocyte and lymphocyte counts exhibit contrasting associations with risk of clinical malaria

Next we sought to determine whether the association between ML ratio and risk of clinical malaria was attributable to the frequency of lymphocytes, monocytes or both cell types. To do this we used both lymphocyte and monocyte count simultaneously as explanatory variables for the negative binomial regression modeling using data from all five cohorts while accounting for multiple sampling of individuals, their age at time of sampling the cell counts and year of survey. If only one cell type was responsible for the association between ML ratio and clinical malaria we would predict that, when both cell counts are used in place of ML ratio for the regression modeling, an association would only be evident with the frequency of the predominant cell type.

Neither lymphocyte count nor monocyte count showed an association with clinical malaria among parasite negative children (Monocyte count, IRR  =  1.1, 95%CI 0.89, 1.35, P  =  0.4; Lymphocyte count, IRR  =  1.0, 95%CI 0.76, 1.39, P  =  0.9). However, among parasite positive children, increased risk of clinical malaria episodes was associated with a high monocyte count (IRR  =  1.7, 95%CI 1.02, 2.73, P  =  0.04) and a low lymphocyte count (IRR  =  0.3, 95%CI 0.19, 0.63, P  =  0.0005). This is in agreement with the high monocyte count and low lymphocyte count observed in previous studies of children presenting to hospital with clinical malaria [14], [16]. The observed pattern of associations was again confirmed using Cox regression with time to first clinical episode as an endpoint among parasite negative children (Monocyte count, HR  =  1.0, 95%CI 0.73, 1.45, P  =  0.9; Lymphocyte count, HR  =  1.0, 95%CI 0.66, 1.48, P  =  0.9) and among parasite positive children (Monocyte count, HR  =  2.4, 95%CI 1.25, 4.72, P  =  0.009; Lymphocyte count, HR  =  0.3, 95%CI 0.14, 0.69, P  =  0.004).

## Discussion

Collectively our data suggests that in the context of an ongoing infection by malaria parasites, the relative frequency of monocytes to lymphocytes in peripheral circulation reflects an individual's capacity to mount an effective immune response. This is not surprising given that monocytes are an essential component of the innate immune response that acts as a link to the adaptive immune system through antigen presentation to lymphocytes. Thus any factors that perturb the function or relative frequency of either cell type could potentially affect an individual's response to infection. So why is the association between ML ratio and risk of clinical malaria only evident among parasite positive children?

Derangement in monocyte or lymphocyte function or frequency is likely to be most evident during an infection, and this may be supported by the observation that ML ratios were consistently higher among parasite positive children in all five cross-sectional surveys (see Table 1). Thus, one possible explanation is that ML ratio is a marker of an individual's capacity to mount an effective immune response against clinical malaria but that it is most informative during an on-going parasite challenge. However, the possibility that presence of asymptomatic parasitaemia at survey merely reflects more frequent exposure to *P. falciparum* that allows greater power to detect associations between ML ratio and clinical malaria needs to be ruled out.

The innate immune response, through pro-inflammatory cytokines such as IFNγ, is thought to contribute to the initial control of parasitaemia following infection by *P. falciparum*, but to also correlate with development of clinical symptoms [24], [25]. This has led to the notion that such a predominantly anti-parasitic immune response requires tempering by the adaptive immune response if effective immunity to clinical malaria is to be achieved. In fact, mounting evidence suggests that the balance of pro- and anti-inflammatory immune responses following exposure to malaria parasites may be an important factor in determining clinical protection [25]–[28]. It is plausible that the ML ratio reflects where in the spectrum of these immune responses an individual is. Hence in the presence of an ongoing asymptomatic infection, a high ML ratio might indicate a predominantly pro-inflammatory immune response that renders individuals susceptible to clinical malaria, but with repeated exposure to *P. falciparum* the adaptive immune response “learns” to produce anti-inflammatory cytokines that effectively temper the pro-inflammatory immune response, leading to a lower ML ratio and prevention of immunopathology. Assessment of the relationship between ML ratio and cellular immune responses is clearly needed if the mechanism underlying the association between elevated ML ratio and susceptibility to clinical episodes of malaria is to be determined.

Our study had several limitations. First, though we used data from children sampled at five annual cross-sectional surveys, both the ML ratios and parasite positive/negative status were measured at a single time-point within any year. Second, monitoring was not done for asymptomatic *P. falciparum* infections hence there is no way of telling whether some children harbored asymptomatic infections in the previous week(s) during monitoring for clinical episodes or shortly before sampling of ML ratios at cross-sectional survey. This is further compounded by the possibility that our microscopy based parasite detection method, which is less sensitive than PCR [22], [23], might have missed some low parasitaemia infections in children classified as parasite negative. The interaction between parasite positive/negative status and ML ratio in predicting risk of clinical malaria is likely to be dynamic and a better understanding of these relationships will be gained from future studies with more frequent sampling of ML ratio and PCR-based monitoring for both asymptomatic *P. falciparum* infections and clinical malaria episodes. Finally, we did not assess the presence of co-infections, either at cross-sectional survey or during monitoring for clinical malaria episodes. Thus, the effect of potential helminth, bacterial or viral co-infections on the relationship between ML ratio and clinical malaria will need to be examined in future studies.

Despite these limitations our results support an association between high ML ratio at recruitment to cross-sectional surveys and increased risk of clinical malaria during follow-up. This observation now needs to be confirmed in other geographic settings.
